# Health Consequences of an Elite Sporting Career: Long-Term Detriment or Long-Term Gain? A Meta-Analysis of 165,000 Former Athletes

**DOI:** 10.1007/s40279-020-01379-5

**Published:** 2020-12-24

**Authors:** Adam Runacres, Kelly A. Mackintosh, Melitta A. McNarry

**Affiliations:** Applied Sports, Technology, Exercise and Medicine (A-STEM) Research Centre, Swansea University, Swansea, UK

## Abstract

**Introduction:**

Exercise is widely accepted to improve health, reducing the risk of premature mortality, cardiovascular disease (CVD) and cancer. However, several epidemiological studies suggest that the exercise-longevity relationship may be ‘J’ shaped; with elite athlete’s likely training above these intensity and volume thresholds. Therefore, the aim of this meta-analysis was to examine this relationship in former elite athletes.

**Methods:**

38,047 English language articles were retrieved from Web of Science, PubMed and SportDiscus databases published after 1970, of which 44 and 24 were included in the systematic review and meta-analysis, respectively. Athletes were split into three groups depending on primary sport: Endurance (END), Mixed/Team, or power (POW). Standard mortality ratio’s (SMR) and standard proportionate mortality ratio (SPMR) were obtained, or calculated, and combined for the meta-analysis.

**Results:**

Athletes lived significantly longer than the general population (male SMR 0.69 [95% CI 0.61–0.78]; female SMR 0.51 [95% CI 0.40–0.65]; both *p* < 0.01). There was no survival benefit for male POW athletes compared to the general population (SMR 1.04 [95% CI 0.91–1.12]). Although male athlete’s CVD (SMR 0.73 [95% CI 0.62–0.85]) and cancer mortality (SMR 0.75 [95% CI 0.63–0.89]), were significantly reduced compared to the general population, there was no risk-reduction for POW athletes CVD mortality (SMR 1.10 [0.86–1.40]) or END athletes cancer mortality (SMR 0.73 [0.50–1.07]). There was insufficient data to calculate female sport-specific SMR’s.

**Discussion:**

Overall, athletes live longer and have a reduced incidence of both CVD and cancer mortality compared to the general population, refuting the ‘J’ shape hypothesis. However, different health risks may be apparent according to sports classification, and between sexes, warranting further investigation.

*Trial registration* PROSPERO (registration number: CRD42019130688).

**Supplementary Information:**

The online version contains supplementary material available at 10.1007/s40279-020-01379-5.

## Key Points

**Table:** 

Elite athletes live longer than the general population.
Sport-specific differences in mortality, and disease, risk may be evident.
More research is needed to examine the impact of an elite sporting career in female athletes with a minimum follow-up period of 30 years.

## Introduction

The benefits associated with regular exercise for physical and mental health in the general population are well-evidenced, with inactivity strongly correlated with an increased risk of premature mortality [1–4]. Indeed, mortality associated with cardiovascular disease (CVD) and cancer, the most prevalent causes of mortality worldwide [5], is exacerbated by physical inactivity [6, 7] and decreased by regular exercise [6, 8]. Specifically, it is suggested that for every one unit increase in maximal metabolic equivalent of task (MET) capacity, the likelihood of CVD mortality is reduced by 15% [6]. Similarly, cancer incidence and mortality rates were 27 and 37% lower in the fittest and least fit group, respectively, in a 16-year longitudinal study of Finnish men [9]. Furthermore, this relationship persisted even after accounting for smoking habits, alcohol intake, waist-to-hip ratio, socioeconomic status and nutritional intake, highlighting the importance of exercise in the prevention of cancer [9].

Despite the benefits associated with regular exercise, there is a body of evidence that suggests the exercise-longevity relationship may be ‘J’ shaped, with exercise beyond certain volume and intensity thresholds detrimental to health [10–15]. Specifically, Mohlenkamp et al. [15] reported that, over a two-year observational period, recreational German marathon runners had a similar incidence of a cardiovascular (CV) event compared to a population with established coronary heart disease (CHD). Furthermore, the Copenhagen Heart Study reported light and moderate joggers to demonstrate lower mortality hazard ratios (0.22 and 0.66, respectively) compared to strenuous joggers (HR 1.97) [14]. Similarly, those who exercised every day in the Million Women study were at an increased risk of a CV event compared to women who had at least one rest day during the week [13].

Elite athletes typically engage in training at levels far exceeding those reported in epidemiological studies, raising questions as to whether elite athletes are potentially at an elevated risk of premature mortality, CVD and/or cancer [10, 12, 13]. Such a concept has received considerable research attention. Indeed, two recent systematic reviews and a meta-analysis investigated the relationship between long-term intensive training, health, and mortality in elite athletes and the general population [16–18]. Taken together, these reviews suggest that elite athletes live longer than the general population and have a lower mortality rate from both CVD and cancer [16–18]. However, these reviews did not stratify by sport type (i.e. aerobic, power, team sports). Consequently, the importance of training types and sporting demands, therefore, largely remains to be elucidated. For example, in comparison to endurance athletes, power (POW) sport athletes have an increased body mass index (BMI) [19, 20], which is an independent risk factor for future CVD [21]. Furthermore, endurance (END) training has been shown to lower several key inflammatory markers [22], which, whilst this remains contentious, could reduce the risk of long-term CVD risk [15, 23].

Therefore, the aim of this systematic review and meta-analysis was to examine the relationship between chronic intensive exercise training and mortality in former elite athletes, according to sport type, in comparison to their non-elite counterparts.

## Methods

### Data Sources, Literature Search and Inclusion Criteria

This systematic review was registered on PROSPERO (registration number: CRD42019130688) and was conducted in accordance with the PRISMA guidelines [24, 25]. The keywords were split into three levels to search scientific databases and were compromised of the following (1) *mortality* or *death* or *longevit*y; (2) *elite* or *athletes* or *Olympic*; and (3) *excessive* or *training* or *chronic exercise*. All keywords were used in combination and different iterations to capture all results, with the full search terms available in the Supplementary Material.

The inclusion criteria for studies in the meta-analysis was: (1) written in the English language; (2) experimental participants were male or female former athletes of at least national standard, with some information on their sporting history provided; (3) the study included a general population reference group; (4) data were reported on mortality, CVD and/or cancer-specific mortality in male or female athletes; (5) data were reported as a standardised mortality ratio (SMR), or standardised proportional mortality ratio (SPMR), with 95% confidence limits, or provided sufficient data (observed/expected mortality) to allow either SMR or SPMR to be calculated; and (6) the studies were of a retrospective, or prospective, methodological design. Any non-peer-reviewed grey literature, including conference papers and theses, were excluded. Moreover, any studies that had a follow-up of ≤ 5 years were excluded, along with studies which reported the primary outcome of mortality but did not use SMR, or the data was not provided to allow this to be calculated. In the case of any disagreements regarding the inclusion of a study that were not able to be resolved (between AR and MM), KM was consulted to reach a consensus, which occurred on five occasions.

**Fig. 1:**
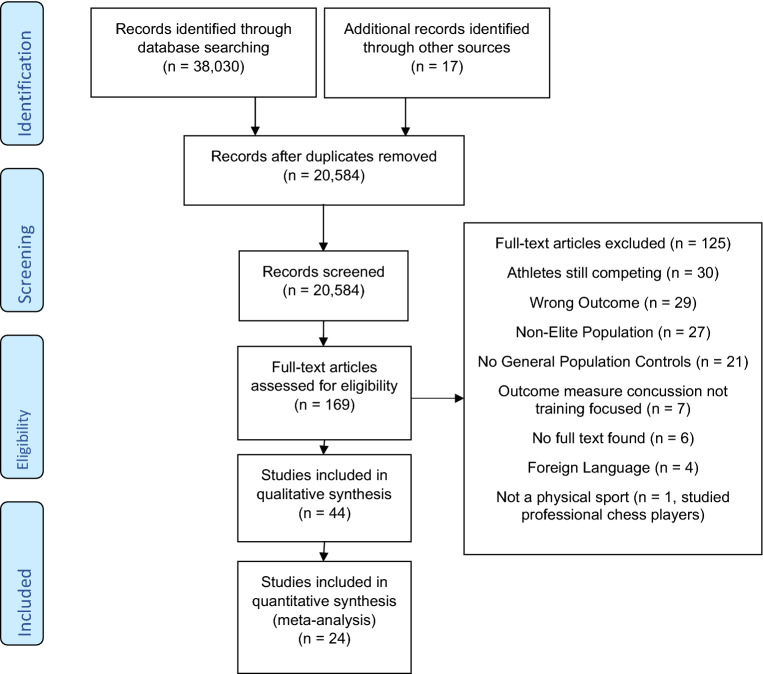
Schematic flow diagram of the systematic review and meta-analysis process

Studies were searched for, and identified, through scientific databases and by scanning the reference list of identified studies. The search was performed in Web of Science (1970–2019), PubMed (1970–2019) and SportDiscus (1970–2019). All potentially relevant studies, including reference lists and abstracts, were compiled in Rayyan QCRI software [26]. Two authors (AR and MAM) then screened all identified titles and abstracts to identify studies for full-text review. From an initial search of 38,047 results, 37,878 were excluded. Consequently, 169 were taken forward for full-text review of which 43 were finally included within the systematic review (Table 1); 24 of which were also appropriate for the meta-analysis (Fig. 1).

**Table 1:** Key information about the studies included within the meta-analysis

References	Number of participants	Average follow-up (years)	All-cause mortality SMR (95% CI)	CVD mortality SMR (95% CI)	Cancer mortality SMR (95% CI)
Sarna et al. [30]	2613 former Finnish athletes 1712 military control participants	44.5	0.92 (0.84–1.01)	0.95 (0.81–1.09)	0.96 (0.75–1.11)
Kettunen et al. [35]	2263 former Finnish athletes 1657 military control participants	50	0.98 (0.91–1.05)	#	0.89 (0.76–1.03)
Lincoln et al. [41]	9778 former NFL players US reference values	18.5	0.46 (0.40–0.52)**	0.68 (0.50–0.90)**	0.41 (0.26–0.62)**
Antero-Jacquemin et al. [36]	2403 (601 female) former French Olympians French population reference values	20.3–43.7	M 0.51 (0.45–0.59)** F 0.49 (0.26–0.85)**	M 0.55 (0.41–0.73)** Insufficient data to compute F SMR	M 0.55 (0.43–0.69)** Insufficient data to compute F SMR
Marijon et al. [42]	786 former Tour de France cyclists French population reference values	32.5	0.59 (0.51–0.68)**	0.67 (0.50–0.88)**	0.56 (0.42–0.72)**
Kontro et al. [31]	900 former Finnish athletes 900 brothers of the Finnish athletes	77.5	1.00 (0.93–1.08)	#	1.47 (1.22–1.73)**
Grimsmo et al. [50]	122 endurance skiers Norwegian population reference values	30	0.78 (0.50–1.05)	Not reported	Not reported
Antero-Jacquemin et al. [37]	203 French Olympic rowers French population reference values	50	0.58 (0.43–0.78)**	0.41 (0.16–0.84)**	0.59 (0.29–1.07)
Menotti et al. [43]	983 (283 female) former track and field athletes	18.6	Overall 0.70 (0.59–0.82)** M 0.73 (0.60–0.86) F 0.48 (0.20–0.76)	Not reported	Not reported
Gajewski and Poznanska [44]	2113 (424 Female) former Polish Olympic athletes Polish population reference values	27	Overall 0.51 (0.48–0.54)** M 0.50 (0.44–0.56)** F 0.73 (0.48–1.05)	Not reported	Not reported
Kujala et al. [32]	2009 former Finnish athletes Finnish population reference values	47.5	0.74 (0.69–0.79)**	0.72 (0.64–0.82)**	^##^
Lehman et al. [38]	3439 former NFL players US population reference values	33.5	0.53 (0.48–0.59)**	0.68 (0.56–0.81)**	0.58 (0.46–0.72)**
Waterbor et al. [51]	958 MLB players US population reference values	59	0.94 (0.88–1.00)	^#^	1.05 (0.89–1.22)
Taioli [45]	5389 Italian footballers Italian population reference values	28	**0.68 (0.52–0.86)**	**0.41 (0.20–0.73)**	**0.31 (0.15–0.55)**
Schnohr [46]	297 former Danish Olympians Danish population reference values	66	0.96 (0.79–1.12)	0.95 (0.67–1.34)	0.94 (0.61–1.44)
van Sasse et al. [52]	2129 former Dutch endurance skaters Dutch population reference population	32	0.76 (0.68–0.85)	Not reported	Not reported
Farahmand et al. [47]	73,622 (24,403 female) endurance ski racers Swedish population reference values	5.5	**Overall 0.48 (0.46–0.51)** **M 0.49 (0.44–0.54)** **F 0.45 (0.40–0.50)**	**Overall 0.43 (0.35–0.51)** **M 0.44 (0.36–0.54)** **F 0.30 (0.11–0.50)**	**Overall 0.61 (0.52–0.71)** **M 0.62 (0.52–0.74)** **F 0.58 (0.41–0.74)**
Radonić et al. [39]	233 Croatian Olympic medallists Croatian population reference values	35	**0.73 (0.56–0.94)**	**0.61 (0.38–0.93)**	0.70 (0.40–1.12)
Baron et al. [33]	3439 NFL players US population reference values	34	**0.53 (0.46–0.72)**	**0.68 (0.56–0.81)**	0.58 (0.46–0.73)
Mackay et al. [40]	7676 former professional footballers 23,028 control participants	18	**0.93 (0.91–0.95)**	^#^	^##^
Nguyen et al. [34]	16,637 former MLB players US population reference values	36	**0.76 (0.73–0.78)**	**0.81 (0.77–0.85)**	**0.80 (0.75–0.86)**
Kalist and Peng [48]	2641 former MLB players US population reference values	20	**0.31 (0.23–0.39)**	Not reported	Not reported
Belli and Vanacore [53]	~ 24,000 Italian footballers Italian population reference values	18	1.00 (0.90–1.10)	0.83 (0.69–1.00)	1.11 (0.97–1.28)
Gadja et al. 2008 [49]	455 deceased polish elite footballers Polish population reference values	–	Not reported	**Under 65–1.29 (0.90–1.68)** **Over 65–1.17 (0.88–1.45)**	Under 65–0.81 (0.45–1.16) Over 65–0.94 (0.55–1.33)

*CI* confidence interval, *SMR* standardised mortality ratio, *NFL* national football league, *MLB* major league baseball, *M* male, *F* female

SMR’s in bold indicate a significant difference between the athletes and the control population (*p* < 0.05). ^#^Kettunen et al. [35], Kontro et al. [31], Mackay et al. [40] all reported specific SMR values on Ischemic Heart Disease (IHD) respectively, and Waterbor et al. [51] reported SMR values for Arteriosclerotic Heart Disease therefore they were removed from CVD analyses as overall CVD mortality was assessed. ^##^Mackay et al. [40] and Kujala et al. [32] report SMR’s for lung cancer specifically and so they were removed from the overall analysis as overall cancer mortality was assessed

### Data Extraction

A data table was created extracting the following information: authors and year of publication, number of participants followed, the primary sport of those athletes (if available), how long the athletes were followed for, all-cause mortality SMR, CVD-specific SMR and cancer-specific SMR. When SMR was not directly reported, it was calculated from the reported observed and expected deaths as SMR = observed (O) death/expected (E) death [18]. If the expected number of deaths was not reported from population data, the number in the referent group was used as the expected value and the SPMR defined as (athlete observed death/number in athlete population)/(control group death/number in the control group). To calculate 95% confidence intervals (CI) for both methods, the formula: SMR *or* SPMR ± (1.96 * standard error of estimate; SEE) defined as √ (O)/E [27] was used. These two metrics are, therefore, uniform and can be combined to create a pooled SMR. The Newcastle–Ottawa Quality Assessment tool [28] was used to assess the quality of each study included within the meta-analysis.

Following the overall risk calculations, specific SMR’s were calculated, where possible, according to sport. Specifically, in line with other research, END activities were defined as any sport requiring more than 10 min of continuous effort [29]. The END sports in the meta-analysis meeting this criterion were: middle- and long-distance runners, rowers, cross-country skiers, ice skaters and tour de France cyclists. A ‘team sport’ was defined as any sport in which the performance is predominantly made up of repeated intermittent efforts [29]. Team sports identified in this meta-analysis were American footballers, baseball players, footballers, ice hockey players and basketball players. Finally, POW sports were defined as any predominantly anaerobic sport [30]. The sports in the POW category for this meta-analysis included: boxers, wrestlers, weightlifters, and throwing events in track and field.

### Statistics

All meta-analyses statistics were performed using *meta*, *metagen* and *metaforest* packages in R Studio (R Studio v1.2.2019, R Studio, Boston, MA) to calculate the pooled SMR and create the subsequent forest plots. Initially, all SMR and SPMR, values, and their 95% CI’s were logged to determine effect sizes on a natural scale and then the SEE was calculated for each study. The resulting data were then run through *metagen*, where the pooled SMR was back-transformed to the original SMR scale. The pooled SMR indicates the risk in athletes compared to the general population, with a value of < 1 indicating a lowered risk, > 1 indicating a greater risk and 1 indicating the same risk. Meta-regressions were also run to establish the relationships between outcome variables and possible confounding factors using the metareg function in R. The pooled SMR was calculated using a random-effects model with heterogeneity assessed using the *I*^2^ and *Q* statistic. Risk of publication bias assessed using a combination of the Egger’s Statistic and funnel plots.

## Results

The total number of athletes included within the 24 studies was 165,033, with 139,322 males (84.4%) and 25,711 females (15.6%). There was insufficient data to split the females by sport type, so this was only done for male athletes. Of the male sample, 78,096 (47.3%) were END athletes, 78,689 (47.7%) were team sport athletes, 3,202 (1.9%) were POW sport athletes, and 5046 (3.1%) of the athletes were Olympians/World Champions where their primary sports could not be established. All included studies were of retrospective methodological design.

The Newcastle–Ottawa scale assesses the methodological quality, and generalisability of an individual study, with higher scores indicating high methodological quality. Of a possible maximum score of 9 on the Newcastle–Ottawa quality score, five, six, nine and four papers scored nine [30–34], eight [35–40], seven [41–49] and six [50–53], respectively. Funnel plots were used to assess publication bias (Supplementary Figures 2–4), with all-cause, CVD, and cancer mortality demonstrating publication bias, indicated by the wide range of log SMRs reported in the included studies.

**Fig. 2:**
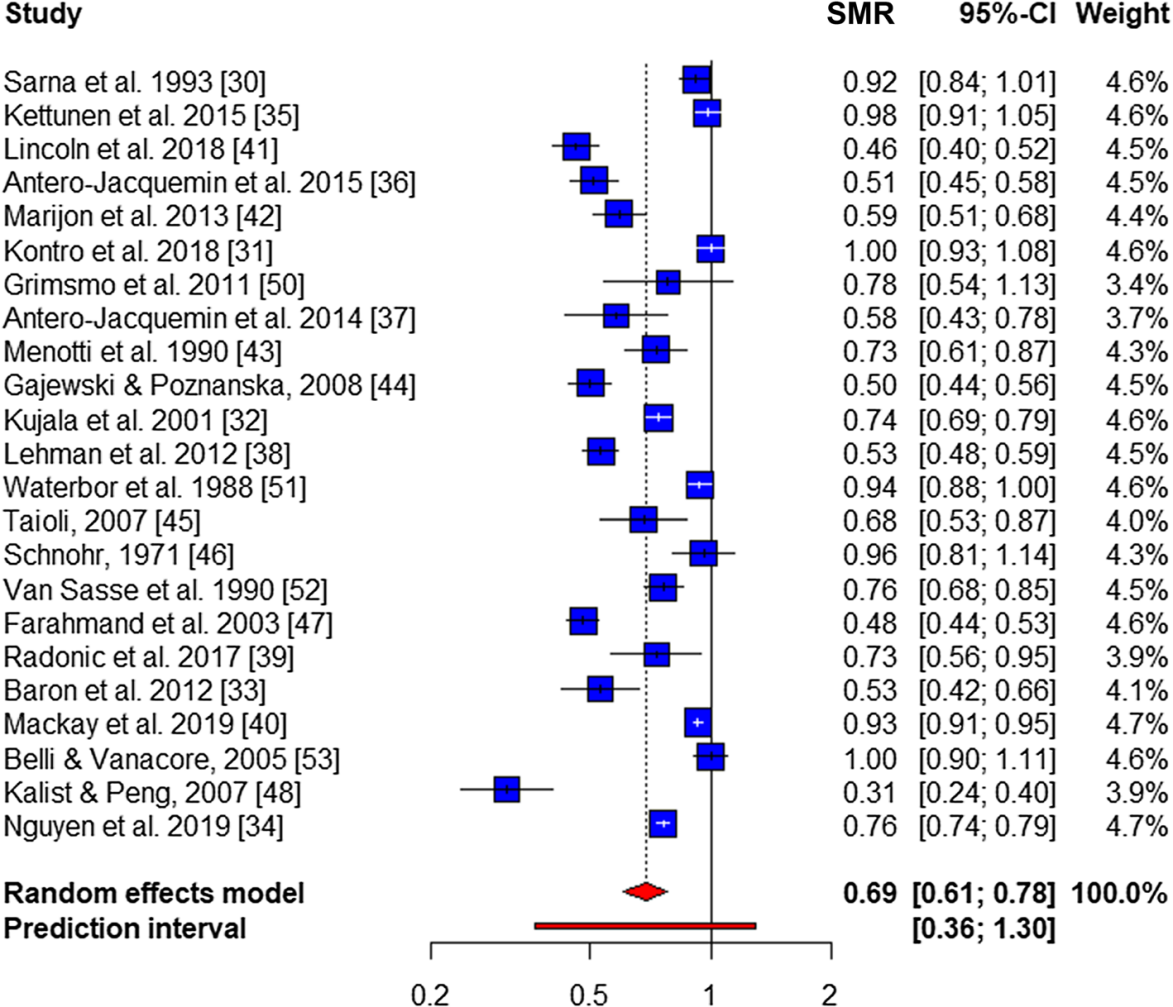
Overall male mortality forest plot

**Fig. 3:**
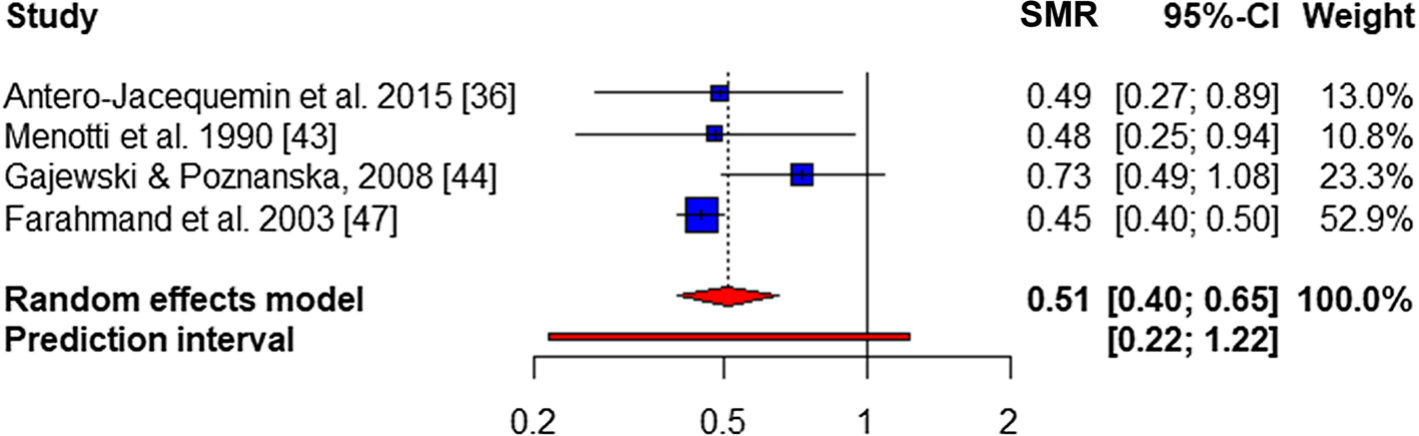
Overall female mortality forest plot

Overall, all-cause mortality in male and female athletes was reported in 23 out of 24 studies (164,833 athletes), creating a pooled SMR of 0.67 (95% CI 0.59–0.75; *p* < 0.01), with some evidence of publication bias [*p* < 0.05 (Supplementary Figure 2)] and significant heterogeneity (*I*^*2*^ = 96.9%; *Q* = 850.7; *p* < 0.01). Sub-group analyses revealed male all-cause mortality was reported in 23 studies [30–48, 50–53] (139,122 athletes; 99.7% of all male athletes), creating a pooled SMR of 0.69 (95% CI 0.61–0.78; *p* < 0.01), with no evidence of publication bias (*p* = 0.07) and significant heterogeneity (*I*^2^ = 97.0%; *Q* = 730.3; *p* < 0.01) (Fig. 2). Female all-cause mortality was reported in four studies [36, 43, 44, 47] (25,711 female athletes; 100%) leading to a pooled SMR of 0.51 (95% CI 0.40–0.65, *p* < 0.01), with no evidence of bias (*p* = 0.41) and no significant heterogeneity (*I*^2^ = 45.1%, *Q* = 5.5, *p* > 0.05) (Fig. 3). There was insufficient data to calculate a meta-SMR for either CVD or cancer mortality in females, therefore, this was only performed in male athletes.

**Fig. 4:**
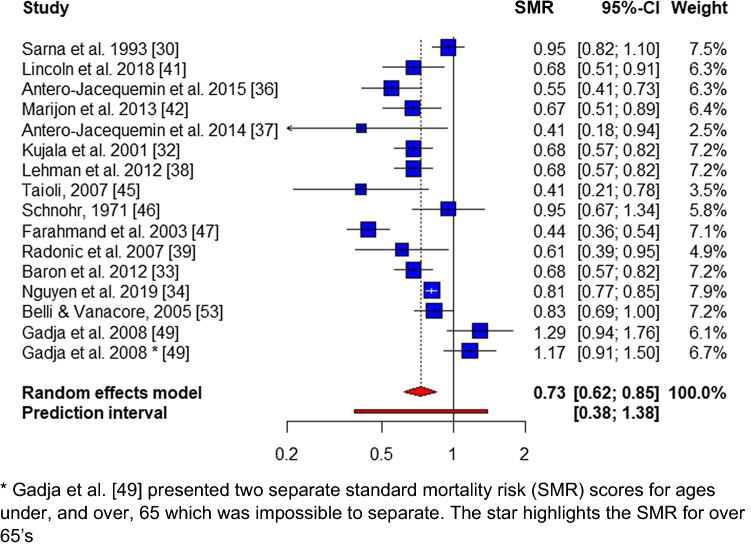
Overall male cardiovascular mortality forest plot

**Fig. 5:**
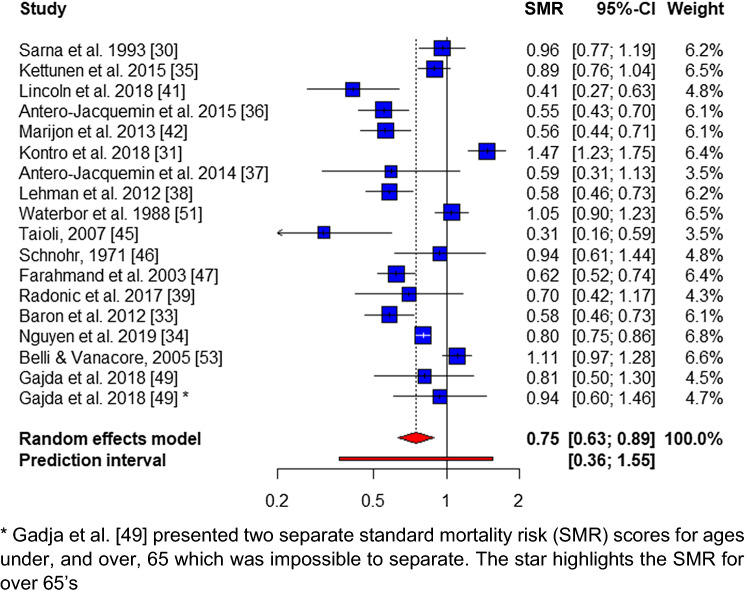
Overall male cancer mortality forest plot

Overall, male CVD mortality was reported in 15 studies [30, 32–34, 36–39, 41, 42, 45–47, 49, 50, 53] (118,288 athletes, 84.8%), demonstrating a pooled SMR of 0.73 (95% CI 0.62–0.85; *p* < 0.01), with no publication bias (*p* = 0.26) and significant heterogeneity (*I*^2^ = 81.8%, *Q* = 82.6, *p* < 0.01) (Fig. 4). Overall cancer mortality was reported in 17 studies [30, 31, 33–39, 41, 42, 45–47, 49, 51, 53], with a pooled-SMR of 0.75 (95% CI 0.63–0.89, *p* < 0.05), no evidence of publication bias (*p* = 0.28) and significant heterogeneity (*I*^2^ = 88.1%, *Q* = 143.1, *p* < 0.01) (Fig. 5).

Endurance and team sport athlete’s all-cause (END: *I*^*2*^ = 98.7%, *p* < 0.01; Team: *I*^*2*^ = 97.0%, *p* < 0.01) and CVD mortality (END: *I*^*2*^ = 96.3%, *p* < 0.01; Team: *I*^*2*^ = 78.0%, *p* < 0.01) was significantly lower than the general population, however, POW athletes’ all-cause (*I*^*2*^ = 77.8%, *p* > 0.81) and CVD (*I*^*2*^ = 84.9%, *p* > 0.46) mortality was not significantly different to the general population. For cancer-specific mortality, both team (*I*^*2*^ = 86.2%, *p* < 0.01) and POW (*I*^*2*^ = 53.3%, *p* < 0.01) athletes pooled-SMR’s were significantly lower than the general population, but endurance athlete cancer mortality was not (*I*^*2*^ = 96.1%, *p* > 0.11). All of the sub-analyses were heterogeneous (*p* > 0.05), with the exception of cancer mortality for power athletes (*p* < 0.05), with no evidence of bias except for team sport all-cause mortality [Eggers test *p* < 0.05 (Table 2)].

**Table 2:** Sport-specific all-cause, CVD and cancer pooled-SMR’s

	Endurance sports				Team sports				Power sports			
	Number of athletes (%)	SMR (95% CI)	Heterogenous (Y/N)	Bias (Y/N)	Number of athletes (%)	SMR (95% CI)	Heterogenous (Y/N)	Bias (Y/N)	Number of athletes (%)	SMR (95% CI)	Heterogenous (Y/N)	Bias (Y/N)
All-cause mortality	53,476 (38.4%)	**0.65 (0.54–0.77)**	Y	N	78,504 (56.4%)	**0.68 (0.57–0.81)**	Y	Y	2826 (2.0%)	1.04 (0.91–1.12)	Y	N
CVD mortality	50,788 (38.4%)	**0.63 (0.44–0.91)**	Y	N	65,078 (55.0%)	**0.76 (0.64–0.92)**	Y	N	1885 (1.6%)	1.10 (0.86–1.40)	Y	N
Cancer mortality	50,511 (38.8%)	0.73 (0.50–1.07)	Y	N	65,280 (53.3%)	**0.73 (0.57–0.93)**	Y	N	1185 (1.5%)	**0.51 (0.35–0.75)**	N	N

*SMR* standardised mortality ratio, *CI* confidence interval, *Y* yes, *N* no

All SMR’s in bold indicate a significant difference compared to the general population (*p* < 0.05). An analysis was deemed heterogeneous based on a combination of the *I*^2^ and *Q* statistics and if the *p* value was ≤ 0.05. An analysis was deemed not biased if the *p* value derived from the eggers test was ≥ 0.05. More detail can be found in the Supplementary Files Online

### Meta-Regression and Sensitivity Analyses

Sensitivity analyses revealed that when (1) the four-lowest quality studies were removed; (2) only studies incorporating athletes actively competing after 1945; or (3) only studies published after 2010 were included, the pooled SMR remained similar to the overall SMR (0.64–0.68). Indeed, meta-regressions demonstrated no significant interaction with SMR for any of the three data constraints. However, when studies with ≤ 30 years follow-up were excluded, the pooled SMR increased to 0.74 (95% CI 0.65–0.84). Moreover, a significant positive association was observed between follow-up length and all-cause (*β* = 0.01, *Z* = 2.94, *p* < 0.01) and cancer mortality (*β* = 0.01, *Z* = 1.93, *p* > 0.05). However, no significant association was reported between follow-up length and CVD mortality SMR (*β* < 0.01, *Z* = 0.90, *p* = 0.36).

## Discussion

This was the first systematic review and meta-analysis to examine sport-specific all-cause mortality in former elite athletes and to consider CVD- and cancer-specific mortality, the two most prevalent diseases worldwide. The key findings from this review are: (1) male and female elite athletes live longer than the general population; (2) male athletes have a lower incidence of CVD and cancer mortality than the general population; (3) power sport athletes all-cause and CVD mortality were not significantly different to the general population; (4) endurance athletes cancer mortality was not significantly different to the general population and (5) increased follow-up length increased the SMR for all-cause and cancer mortality, but not CVD. Furthermore, there is currently insufficient data to allow sport-level comparisons for female athletes.

### All-Cause Mortality

Over recent years, an argument has been made that chronic, intensive exercise may be harmful to health [10–12] and lead to a greater chance of premature mortality, or an increased incidence cardiovascular events [14, 15, 54]. However, the current evidence refutes these arguments; male and female athletes had a 31 and 49% lower risk of all-cause mortality than the general population, respectively. This seems to indicate that the female survival advantage (females are expected to live 6–8 years longer than males at birth [55]) persists, and is even extended, after a career in elite sport. However, female mortality was only explored in 25,711 athletes, 24,403 (94.9%) of which were identified from a single study [47], hence there was no significant heterogeneity within the pooled-SMR generated. Therefore, more research in female athletes is needed to confirm the survival benefit in highly active female athletes. Moreover, more research including a follow-up period of ≥ 30 years are needed given the positive association between reduced survival estimates and follow-up time.

Given that the standardised mortality ratio was the most common method of reporting the risk of mortality in elite athletes, this method was chosen for the meta-analysis. However, life expectancy and age at death in male athletes has also been explored. Specifically, Clarke et al. [56] reported an average 2.8 year survival advantage in a cohort of 15,174 Olympic athletes from nine countries, with a cohort study of 2814 French Olympians gaining an average of 6.5 years [29]. These results are, therefore, largely in accord with those of the current meta-analysis, as the lowered SMR risk indicates a longer survival in former elite athletes compared to the general population.

Despite the apparent survival benefit of elite athletes, one common and important criticism of the literature is the applicability of comparing former elite athletes to the general population. Elite athletes may be characterised by healthier lifestyles post-retirement than the general population and engage in more leisure-time physical activity (LTPA), both of which predict all-cause mortality [31, 44, 57, 58]. It is, therefore, not currently possible to distinguish the influence of intensive training per se from overall lifestyle factors. Indeed, it may be worth noting that when Sarna et al. [30] and Kettunen et al. [35] used a control group formed of military fit personnel, the SPMR was not significantly different relative to elite athletes (0.92 and 0.98, respectively). Additionally, some studies have only reported survival benefits up to a specific age, rather than across the whole lifespan [40, 46, 49]. Specifically, Schnohr [46] found that athletes up to 50 years had a SMR of 0.61, with athletes aged over 50 and 65 years having SMR’s of 1.08 and 1.02, respectively. Similarly, former Scottish footballers only had a survival benefit up to the age of 60 years [40], with Polish footballers having a benefit until 75 years [49], after which the mortality was the same or greater than the general population. Conversely, Antero-Jacquemin [29] reported an increased longevity in French Olympians after 50 years of age, thus, it is unknown why this apparent loss of survival advantage occurs, in some, but not all, athletes. Further work is needed to elucidate the potential mechanisms.

### Sport-Specific Mortality

Male END athletes had the most favourable all-cause mortality rate and lived significantly longer than the general population (SMR 0.65). Indeed, Clarke et al. [56] reported a 13% greater survival benefit for medallists in endurance sports, with similar benefits reported in marathon runners (+ 4.3 years [59]), tour de France cyclists (+ 8 years [60]) and Olympians involved in endurance sports (+ 6.3 years [29]). Endurance athletes have consistently been shown to have favourable mortality compared to the general population, attributed to an increased cardiorespiratory fitness (CRF) and subsequent maintenance of CRF throughout the lifespan. Specifically, every 1 MET increase in maximal capacity reduces the likelihood of all-cause mortality by 15% [6]. Furthermore, the difference is unlikely to be explained by genetic factors as it has recently been shown that elite athletes who undertake strenuous aerobic exercise exhibit similar disease-trait-related genotypes to the general population [61]. Thus, endurance athletes are still predisposed to similar levels of disease to the general population.

Male team sport athletes, the biggest sub-group within the meta-analysis including 78,504 (56.4%) of all male athletes, also demonstrated a favourable all-cause mortality (SMR 0.68). However, it must be noted that significant bias was evident (Eggers statistic *p* = 0.01) and so these results should be interpreted with caution. This may be explained, at least in part, by two studies in the team sport meta-analysis including athletes competing before 1915 [46, 51]. Specifically, sporting practices, training demands, athlete welfare and advances in health care make it difficult to directly compare across such a large time-span and gain reliable results. Nevertheless, a large body of research in North American sports report a survival benefit in former baseballers (+ 4 to 5 years [62–64]), American football players (+ 6.1 years [65]) and basketballers (+ 4.3 to 5.5 years [66]), but the same was not observed in footballers (− 1.9 years [67]). It should be acknowledged, however, that three of these studies, conducted by Abel and Kruger [62, 63, 65], also involved athletes who made their professional debuts before 1940, so the applicability of their findings to a modern population is questionable. Furthermore, Kuss et al. [67] failed to account for world war deaths, confounding conclusions and potentially explaining the reduced survival incidence reported. Nevertheless, despite these methodological limitations, they advance our understanding, although the generalisability of their results remains questionable and conclusions must be drawn with caution.

Power sport athlete’s all-cause mortality was not significantly different from that of the general population (pooled-SMR 1.04), however, this analysis was only conducted in two studies with participants totalling 2826, or 2.0%, of the overall population. Similar patterns are evident in other studies, with male discus throwers (− 0.6 years) and 100 m runners (− 0.9 years) experiencing marginal premature mortality [59]. In contrast, Clarke et al. [68] reported a modest survival benefit in power athletes, albeit of only 5%. Former Olympic male wrestlers have also been reported to live 13.0 ± 18.4 years longer, although this must be interpreted with caution given that the standard deviation spans 0, indicating some have a premature mortality, and the relatively small sample size included within this study (*n* = 341) [69]. However, contradicting the negative associations of all-cause mortality and power sports, Antero-Jacquemin et al. [29] reported power athletes gained an average of 7.2 years, suggesting a significantly longer life-span. Given the disparity of results across the literature and the small statistical power within this meta-analysis, more research is needed to fully elucidate the long-term effects of competing in power sports.

### Cardiovascular Disease Mortality

Overall, the pooled-SMR risk of CVD mortality (0.73) was significantly lower than the general population. This is not surprising given the long-established relationship between CRF and CVD mortality [70]. Specifically, men in the highest quintile of fitness, compared to those in the lowest, had a relative risk of 0.22 (0.12–0.39) for CVD mortality [71], and as little as 1 ml⋅kg^−1^⋅min^−1^ increase in CRF decreased the risk of CVD mortality by 9% [72]. Cardiorespiratory fitness is critical for most END and team sports athlete’s performance and, consequently, these athletes occupy the top percentile for CRF values and present the lowest risk (SMR 0.63 and 0.76, respectively). Thus, the superior CRF and consequent lower CVD mortality risk in team and END athletes is one of the main reasons suggested for the observed increased longevity in END and team athletes [16, 29], and the lack of protective effect in power athletes (SMR 1.10).

Four studies [31, 35, 40, 51] were not included within the meta-analysis for CVD mortality as they reported a SMR value for the specific CVD of ischemic heart disease (IHD) [31, 35, 40] or arteriosclerotic heart disease (AHD) [51]. Including specific CVD SMR’s, as opposed to overall CVD risk, could have induced bias and so the decision was made to remove them. However, the SMR for IHD was not significantly different in former Finnish athletes (SMR 0.95 (0.81–1.09) [31]; SMR 1.00 (0.86–1.14) [35]) or former Scottish footballers (SMR 0.91 (0.87–0.96) [40]), in relation to the general population. Similarly, AHD mortality risk was not significantly different in 958 former baseball players (SMR 1.10 (0.99–1.22)) [51]. Furthermore, a recent study in French Olympians reported END athletes are at an increased risk of mortality due to CVD, cumulating in a loss of 1.6 (4.8, 1.2) years [29]. Taken together, these results indicate that END and team athletes may be protected against some, but not all, CVDs. This may, at least in part, explain the overall protective effect of exercise but the minimal impact on IHD and AHD. However, more research is needed to confirm this hypothesis and to establish whether intensive training lowers the specific risk profile and aetiologies of individual CV diseases.

Power athletes pooled-SMR was not significantly different to that of the general population for CVD (SMR 1.10), however, caution is warranted when interpreting this finding as only 1885 male athletes (1.2%) from two studies [30, 32] were included. Nevertheless, American football linemen, who share a lot of characteristics with power sport athletes, had a two to threefold increase in CVD mortality compared to counterparts in other positions [33, 41]. One possible explanation is the increased likelihood of hypertension in power athletes [32, 73, 74], a long-established independent CVD risk factor. Additionally, power sport athletes characteristically have a higher BMI and a relationship between playing/competing time BMI and CVD mortality has been observed [33]. Specifically, American football players who had a playing time BMI of ≥ 30 kg⋅m^2^ had twice the risk of CVD mortality (SMRs 2.02–2.07), compared to those with a BMI ≤ 29.9 kg⋅m^2^ [33]. This risk could be further exacerbated as over a 30-year period power athletes reportedly gained an average of 12.8 kg [75]. So, a question remains as to whether playing-time BMI is the primary risk factor of CVD or subsequent weight-gain post-retirement is a greater indicator of CVD mortality in power athletes.

### Cancer Mortality

Cancer mortality (SMR 0.75) was significantly lower in athletes than the general population. Likewise, elite French athletes had a significantly lower incidence of cancer mortality, gaining an average of 2.3 (1.9–2.6) years [29]. One possible explanation is that former athletes smoke less, drink less, and engage in more LTPA than the general population [31, 57, 58, 76], all of which significantly contribute to cancer risk and mortality. Indeed, Sourmunen et al. [76] reported that when LTPA, smoking status, years of smoking and alcohol consumption were accounted for, there was a minimal protective effect on cancer incidence (standardised incidence ratio 0.89 (0.81–0.97)). Moreover, Pukkala et al. [77] reported that elite athletes had a slightly elevated incidence of non-smoking related cancers (SIR 1.10), with other studies reporting lung cancer mortality was significantly reduced in athletes [32, 40]. This confirms the importance of accounting for lifestyle-related habits when assessing cancer incidence/mortality in this population. This may explain, at least in part, why some populations of footballers [40, 49, 53, 78], Olympians [39, 46] and baseballers [51] all have similar rates of cancer incidence and mortality to the general population, whilst others demonstrate a reduced risk [33, 34, 42, 45].

Endurance athletes’ risk of cancer mortality was not significantly different from the general population (SMR 0.73 (0.50–1.07)). Despite this, END athletes have consistently been found to have favourable longevity compared to the general population and, indeed, other athletes [29, 37, 41, 42, 47, 52]. Thus, it is worth considering whether the non-protective effect on cancer mortality derives from END training, or simply that athletes are living longer and, therefore, have a greater chance of developing cancer. Whilst distinguishing these factors may be challenging, it deserves consideration given that it could alter the interpretation of the results presented and future study directions. Regardless of their increased longevity, however, END athletes are still at a decreased risk of CVD mortality, suggesting the benefit of training is maintained throughout the life-span.

### Limitations

Whilst there are numerous strengths, there are limitations to this review that require consideration. Specifically, not all the athletes within these studies were elite and of national standard, although they were all considered to be highly trained. Moreover, inferences are not able to be made about the specific training that athletes should undertake as such data was rarely reported. As such, conclusions regarding the long-term effects of participating in specific types of training regimes, such as HIIT, resistance or strength training, are precluded. Furthermore, the small number of studies included within the POW athlete sub-group, and female athletes potentially limits the generalisability of these results. It is also noteworthy that some sports may have been mis-classified in previous research (for example, Sarna et al. [30] classified boxing as a power sport), which could have influenced the meta-analysis results. Finally, no inferences can be made as to the relative contribution of lifestyle on overall mortality. Thus, it is hard to distinguish whether any survival benefit observed is because of training, lifestyle choices, or most likely, a combination of both.

## Conclusions

The main conclusions from this review are: (1) overall, male and female athletes’ all-cause mortality is significantly lower than the general population; (2) sub-group analyses revealed END and team sport athletes, but not POW athletes, had a reduced all-cause mortality; (3) POW athletes were at a similar risk of CVD mortality compared to the general population, and; (4) END athletes cancer mortality was not significantly different to the general population. However, more research is warranted in female and power athletes, with a follow-up of ≥ 30 years, to ascertain the long-term benefits/consequences of chronic intensive exercise training in these populations.

## Supplementary Information

Below is the link to the electronic supplementary material.

Supplementary file1 (DOCX 190 kb)

## Declarations

### Availability of data and material

The data that support the findings of this study are available from the corresponding author upon reasonable request.

### Code availability

The code used to within this review is available from the corresponding author upon reasonable request.
