# Supplementary material for: Health Consequences of an Elite Sporting Career: Long-Term Detriment or Long-Term Gain? A Meta-Analysis of 165,000 Former Athletes
Source: Sports Med. 2020 Dec 24;51(2):289–301. doi: 10.1007/s40279-020-01379-5 (PMC7846545; doi:10.1007/s40279-020-01379-5)
Supplement: Supplementary file 1 — Supplementary file1 (DOCX 190 kb) [file 40279_2020_1379_MOESM1_ESM.docx]

**Article Title:** **Health consequences of an elite sporting career – long-term detriment or long-term gain? A Meta-Analysis of 165,000 Former Athletes**

**Journal Name:** Sports Medicine

**Authors:** Runacres, A.^1^, Mackintosh, K.A.^1^, & McNarry, M.A.^1^

**Affiliation:** ^1^ Applied Sports, Technology, Exercise and Medicine (A-STEM) Research Centre, Swansea University, United Kingdom

**Email Address of Corresponding author:** K.Mackintosh@swansea.ac.uk

**Appendix 1**

Full Search Terms

**TOPIC:** (Athlet* OR Players OR Sportsmen OR Sportswomen OR Elite OR Olympic OR Professional, OR World Class)

*AND*

**TOPIC:** (Health OR Mortality OR Death OR Life Expectancy OR Longevity AND Athletes OR Mortality AND Athletes OR Cardiovascular Disease OR CVD OR Cardiovascular disease AND athletes OR CVD AND athletes OR Cancer OR Cancer AND athletes)

*AND*

**TOPIC:** (Training OR Sports OR Competition OR Excessive OR Chronic Exercise OR Chronic Exercise Training OR Chronic Exercise Exposure)

**Appendix 2**

Funnel Plot of All-Cause Mortality


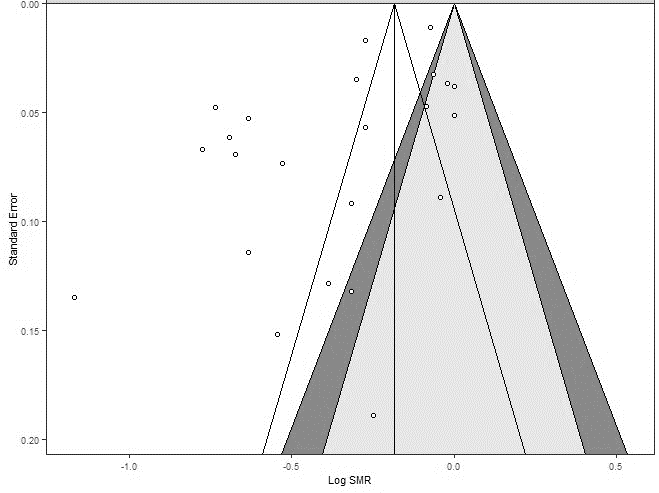


**Appendix 2 –** A funnel plot of the log standard mortality ratio for all-cause mortality versus the logged standard error for all 23 studies included. The light grey and dark grey areas depict significance of p < 0.05 and p < 0.01, respectively.

**Appendix 3**

Funnel Plot of Cardiovascular Mortality


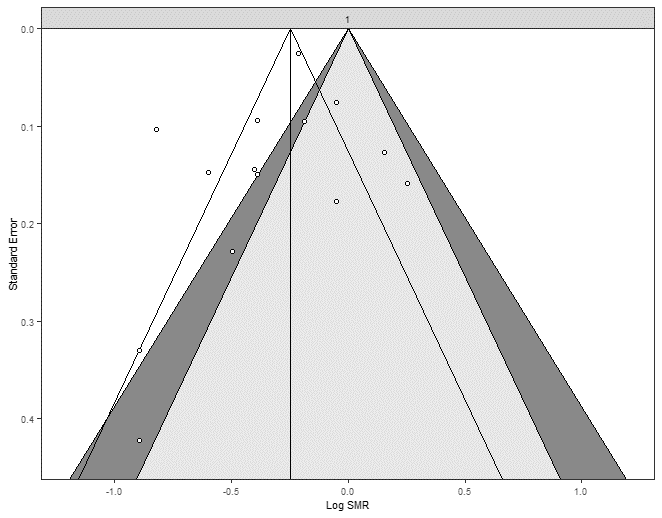


**Appendix 3 –** A funnel plot of the log standard mortality ratio for cardiovascular disease mortality versus the logged standard error for all 15 studies included. The light grey and dark grey areas depict significance of p < 0.05 and p < 0.01, respectively.

**Appendix 4**

Funnel Plot of Cancer Mortality


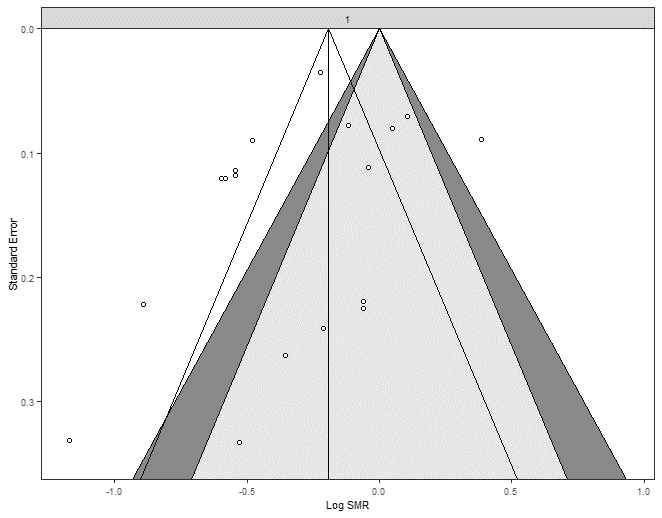

**Appendix 4 –** A funnel plot of the log standard mortality ratio for cancer mortality versus the logged standard error for all 17 studies included. The light grey and dark grey areas depict significance of p < 0.05 and p < 0.01, respectively.
